# Enhancing Bioactive Antioxidants’ Extraction from “Horchata de Chufa” By-Products

**DOI:** 10.3390/foods7100161

**Published:** 2018-10-01

**Authors:** Elena Roselló-Soto, Francisco J. Barba, Predrag Putnik, Danijela Bursać Kovačević, Jose M. Lorenzo, Yara Cantavella-Ferrero

**Affiliations:** 1 Nutrition and Food Science Area, Preventive Medicine and Public Health, Food Sciences, Toxicology and Forensic Medicine Department, Faculty of Pharmacy, Universitat de València, Avda. Vicent Andrés Estellés, s/n, Burjassot, 46100 València, Spain; eroso2@alumni.uv.es (E.R.-S.); Francisco.Barba@uv.es (F.J.B.); yacanfe@alumni.uv.es (Y.C.-F.); 2 Faculty of Food Technology and Biotechnology, University of Zagreb, Pierottijeva 6, 10000 Zagreb, Croatia; 3 Centro Tecnológico de la Carne de Galicia, rúa Galicia No. 4, Parque Tecnológico de Galicia, San Cibrao das Viñas, 32900 Ourense, Spain; jmlorenzo@ceteca.net

**Keywords:** horchata de chufa, tiger nuts, by-products, antioxidant capacity, flavonoids, phenolic compounds

## Abstract

During the production of a traditional drink produced from the tubers of *Cyperus esculentus* L. also known as “horchata de chufa,” a high quantity of by-products are generated. These by-products are rich with valuable biological compounds, hence, there is a need to report their extraction conditions for further use in food production as raw materials. Therefore, the objective of this study was to evaluate and improve the conventional extraction process, applied for recovery of phenolic compounds, total flavonoids, and total antioxidant capacity from the by-products. Independent variables for extraction were: (i) Solvent type (mixtures of ethanol-water (*v*/*v*) at 0%, 25% and 50%); (ii) temperature (40, 50 and 60 °C), and (iii) extraction time (1, 2 and 3 h). The obtained results showed that solvent type, temperature, and time significantly influenced (*p* < 0.05) all investigated parameters. The highest content of total polyphenols (16.02 mg GAE/100 g of dry matter; d.m.), and total flavonoids (30.09 mg CE/100 g d.m.) was achieved by ethanol at 25% (*v*/*v*), after 3 h of extraction with temperatures of 60 °C and 50 °C, respectively. The highest value of antioxidant capacity (1759.81 µM Trolox equivalents/g d.m.) was observed with 50% aqueous ethanol (*v*/*v*), at 60 °C, and 3 h of extraction. From the obtained results, it can be concluded that the by-products of “Horchata de Chufa” are an important source of antioxidant bioactive compounds.

## 1. Introduction

“Horchata de chufa” is a typical refreshing beverage from Spain, having a milky appearance, white color, and pleasant flavor. This drink is obtained from the tubers of the tiger nuts (*Cyperus esculentus* L.). The industrial production of horchata is of great economic importance for the Spanish food industry, and in particular for Valencian Community, representing an annual consumption of ≈50 million liters per year or having an estimated retail value of 60 million euros [1,2]. During the production, a high quantity of by-products is generated, which can represent up to 60% of the raw material [3].

Due to large production of by-products in the food industry [4,5,6], measures have been established for their waste management with the aim to reduce the environmental footprint and to promote further uses in other industrial processes as sustainable raw materials [7]. That is why the scientific community have shown an increased interest in horchata by-products [8].

By-products from the production of “horchata de chufa” can be separated into solid and liquid phases that have suitable compositions for different uses. Due to high content of dietary fiber, antioxidants, and bioactive compounds (polyphenols), the solid residue can be used for functional food production and tackling problem with widespread nutritional deficiencies [9]. Liquid by-product stands out due to its high content of prebiotics and antioxidant compounds, with good technological properties. For instance, water retention and emulsification capacities, while being adequate water-substitute for meat industries [3]. On the down side, Sánchez-Zapata et al. indicated presence of high microbial load in tiger nuts, hence it is necessary to carry out a heat treatment before their addition to the food products [10].

One of the most common methods of processing in food industry is conventional extraction (CEx) by solvent systems, e.g., liquid–liquid or solid–liquid [8]. The CEx involves the use of organic solvents, many of them toxic, such as methanol, hexane or acetone. They potentially pose risks to public health and the environment [11,12]. Luckily a large number of acceptable solvents exist (e.g., ethanol), that are very suitable for the food production [13,14]. According to Mokrani and Madani [15], acetone is the best solvent for the extraction of proanthocyanidins and tannins, whereas ethanol is more efficient for the extraction of flavonoids and their glycosides [16]. Even though methanol is appropriate for extracting phenolic acids and catechin, it is a toxic solvent.

Polyphenols constitute a large group of bioactive compounds (BAC) [17,18]. For example, flavonoids are divided into different subgroups depending on the degree of oxidation of the carbon rings, the main ones being flavonols, flavones, isoflavones, flavan-3-ols, flavanones, and anthocyanins [19]. In the group of non-flavonoids are phenolic acids, water-soluble tannins and stilbenes, with phenolic acids being the most common in dieting [20].

Although a large amount of tiger nuts are produced each year, optimization of a CEx to recover antioxidant BACs for industrial purposes from this matrix is rather scarce. Therefore, this study aimed to evaluate the influence of the solvent type, temperature, and time for recovery of the BACs from by-products remained after production of “horchata de chufa.”

## 2. Materials and Methods

### 2.1. Chemical and Reagents

Acetone, sodium carbonate (Na_2_CO_3_), and sodium hydroxide (NaOH) were purchased from J.T.Baker, Deventer, Netherlands. ABTS (2,2′-azino-bis (3-ethylbenzothiazoline-6-sulfonic acid)), aluminum chloride (AlCl_3_), catechin, ethanol p.a. (99.5%), Folin-Ciocalteau 1 N, gallic acid, sodium nitrite (NaNO_2_), potassium persulfate (K_2_S_2_O_8_) and Trolox (6-hydroxy-2,5,7,8-tetramethylchroman-2-carboxylic acid) were obtained from Sigma-Aaldrich, Steinheim, Germany. Deionized H_2_O was purchased from Millipore, Bedford, MA, USA.

### 2.2. Samples

Tiger nuts (*Cyperus esculentus* L.) were purchased from a local supermarket in Valencia (Spain), with the designation of origin “Chufa de Valencia.” Analyzed by-products in this study were obtained during the laboratory production of “Horchata de Chufa.” Laboratory scale equipment was used to acquire the beverage. For this purpose, 250 g of tiger nuts from Valencia, previously soaked for 8 h, were weighed. Then, the container was filled with 1 dm^3^ of distilled water, the filter vessel was placed, and the tiger nuts were poured. The hand blender was introduced and a slight pressure was applied with the mortar, after pressing the by-products that remained and were subsequently used.

In order to obtain a dehydrated by-product, it was placed in glass trays of a drying oven for 24 h at a constant temperature of 100 °C. Next, the percentage of moisture was determined by the loss of weight due to the evaporation of the water. The procedure was as follows: The porcelain crucibles were placed in the oven for 1 h. Then, it was taken out and left to cool in the desiccator for 20 min, and weighed. On analytical balance, 5 g of the by-products was weighed and placed in different crucibles, then dried in the oven at 100 °C for 24 h. After this, the crucibles were placed inside the desiccator for 20 min until a constant weight. The same procedure was used with fresh tiger nuts with two repetitions for each examination. The moisture was calculated by following Equation (1):

(1) $$\mathrm{Moisture}=\frac{\left(M1-M2\right)}{M}\times 100$$

where: M1 = weight of the crucible with wet sample; M2 = weight of the crucible with dry sample; M = sample weight.

### 2.3. Extraction

For the determination of the total phenolic compounds, total flavonoids and total antioxidant capacity, a solid–liquid extraction was carried out. Firstly, 1 g of dehydrated by-product was weighed and 15 mL of the ethanol:water mixtures were added at different concentrations (0%, 25% and 50%, *v*/*v*), according to a previously established protocol [21]. Then, the beakers with the samples were placed and stirred on a plate with magnetic stirrer. The speed (RPM) was adjusted, to avoid spilling during the stirring. The samples were covered with aluminum foil to avoid the evaporation of the solvent during the extraction. In each of the stirring plate’s rows, the temperature was adjusted to 40, 50 and 60 °C. These conditions were set for all extractions with varying the extraction time (1, 2 and 3 h). The obtained samples (*n* = 27) were filtered and used for determination of the total phenolic compounds (TPC), total flavonoids (TF), and the total antioxidant capacity (TEAC).

### 2.4. Determination of Total Phenolic Compounds (TPC)

To determine the TPC, a previously established method was used with some modifications [22], originally reported by Singleton et al. [23]. Briefly, 0.5 mL of extract was mixed with 4.5 mL of distilled water and afterwards 1 mL of the 2% sodium carbonate solution (*w*/*v*), and 0.25 mL of the Folin-Ciocalteau reagent (1 N) were added. The mixture was left to stand for 1 h at room temperature in darkness. Subsequently, the absorbance was measured at 765 nm. The determination of TPC was performed by interpolating the values in a calibration line of the gallic acid standard (10 µg/mL) at different concentrations between 0–5 µg/mL. The results were expressed as mg equivalents of gallic acid (GAE)/100 g of dry matter. All spectrophotometric measurements were conducted on a Perkin-Elmer Lambda 2 UV/Vis spectrophotometer (Perkin-Elmer, Jügesheim, Germany). All determinations were carried out in duplicates.

### 2.5. Determination of Total Flavonoids (TF)

For TFs determination, the protocol established by Jara-Palacios et al. was used, with some modifications [24]. The AlCl_3_ method used consisted of the formation of chelates with ortho-dihydridoxylated, 3-hydroxylated and 5-hydroxylated flavanoids in basic medium. These chelates have a pink coloration that can be determined spectrophotometrically quantified at λ = 510 nm. In the analysis, 5 mL of diluted extract (1:5, *v*/*v* or 1:25, *v*/*v*) was mixed with 5 mL of distilled water, and 1 mL of 5% NaNO_2_ (*w*/*v*). Then, the closed mixture was left to stand at room temperature for 5 min. After this time, 1.5 mL of 10% AlCl_3_ (*w*/*v*) was added, and the mixture was left at the room temperature for an additional 5 min. Afterwards, 5 mL of 1 M NaOH was added, and the mixture was brought up to 25 mL with distilled water. Then, the absorbance of the mixtures was measured with a wavelength of λ = 510 nm. The concentration of TF was established by calibration line prepared with the catechin standard (50 µg/mL), in concentrations between 0 and 10 µg/mL. The results were expressed as mg equivalents of catechin (CE)/100 g of dry matter.

### 2.6. Determination of Total Antioxidant Capacity (TEAC Method)

The total antioxidant capacity was measured by the modified TEAC method (Trolox equivalent antioxidant capacity) according to the protocol established by Roselló-Soto et al. [22]. The radical ABTS (2,2′-azino-bis(3-ethylbenzothiazoline-6-sulphonic acid) was obtained by mixing 25 mL of 7 mM ABTS with 440 µL of 140 mM potassium persulfate (K_2_S_2_O_8_). The mixture was left to stand at the room temperature for 12–16 h in darkness, while being stable for two days. For the preparation of the working solution, the radical ABTS was diluted with ethanol, until its absorbance was 0.70 ± 0.02 at 734 nm. Then, 2 mL of the working ABTS solution was added to the cuvette and the initial absorbance (A_0_) was measured at a wavelength of λ = 734 nm. When the absorbance values were in required range, then, 0.1 mL of diluted extract (1:2, 1:5, and 1:10 *v*/*v*) was added, and the absorbance was measured at 20 min (A_f_). To calculate the inhibition percentage (%) of the samples, the following equation was used:

% Inhibition = (1 − A_f_/A_0_) × 100 (2)

where A_0_ is the absorbance at initial time and A_f_ is the absorbance obtained after 20 min.

The results were expressed as micromolar (µM) Trolox equivalent (TE)/g of dry matter. In order to obtain the values in µM TE/g of dry matter for the samples, the values of inhibition percentage were interpolated in a previously prepared calibration curve. This was done by using different concentrations of a Trolox^®^ standard as *x*-axis, and the percentage of inhibition for these Trolox concentrations as *y*-axis.

### 2.7. Statistical Analyses

For the study of significant differences in the results obtained, an analysis of variance (ANOVA) was carried out. Pearson’s correlations measured associations among the total antioxidant capacity, total phenolic compounds, and total flavonoids according to previous research [25]. For all statistical data, *p* < 0.05 was considered statistically significant. The statistical analysis was performed with the Statgraphics Centurion XVI^®^ program (StatPoint Technologies, Inc., Warrenton, VA, USA).

## 3. Results and Discussion

### 3.1. Total Phenolic Compounds (TPC)

**Figure 1:**
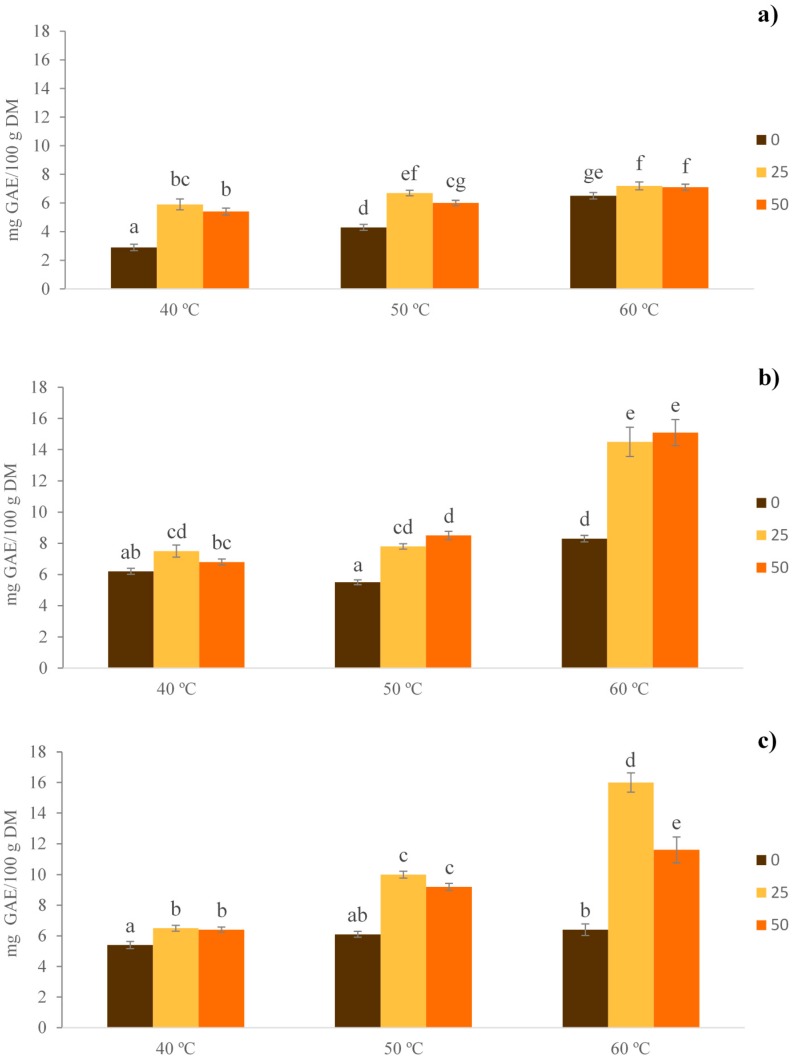
Content of total phenolic compounds, after extraction with different hydroethanolic mixtures (0%, 25% and 50% ethanol:water, *v*/*v*); temperature (40, 50 and 60 °C); and extraction time. (**a**) 1 h; (**b**) 2 h; and (**c**) 3 h. Different letters show statistically significant differences (*p* < 0.05) for temperatures and concentrations of ethanol used. DM: dry matter.

The TPC in the extracts obtained under different extraction conditions (i.e., temperature, time and different percentages of ethanol:water) was evaluated. From Figure 1 it can be seen that TPC values ranged from 2.86 to 16.02 mg GAE/100 g of dry matter, and were dependent on the temperatures, times, and concentrations of used ethanol. All of these three factors had significant effect (*p* < 0.05) on the content of TPC from extracts.

The highest value of phenolic compounds (16.02 mg GAE/100 g of dry matter) was observed at 60 °C with 25% (*v*/*v*) of ethanol in water, and an extraction time of 3 h. In fact, seen in Figure 1, the extraction efficiency for TPC increased with higher temperatures, judging by the values of 7.50, 7.80, and 14.46 mg GAE/100 g of dry matter, at 40, 50 and 60 °C, respectively. All of these were measured at the same concentration of ethanol and extraction time (25% ethanol, 2 h). These results may be explained by the increase in the solubility of phenolic compounds at higher temperatures with increased speed of diffusion which favors mass transfer phenomena [15,26]. However, this is outweighed by the polyphenolic thermal stability, as after maximum increases of temperature, their thermal degradation will occur in numerous plant material [26,27,28,29,30].

In a similar study by Yuan et al., for hazelnut shells, higher TPC was found with increased temperature, i.e., for 30 °C and 50 °C that had values of 801 mg and 1050 mg GAE/100 g of hazelnut shell, respectively [26]. This indicated that as the temperature increases, the surface tension and the viscosity of the solvent decreases, thus, favoring the inclusion of the solvent to matrix. Dorta et al. suggested that higher temperatures can affect antioxidant activity and reduce the stability of the phenolic compounds contained in the extracts [31]. On the contrary, Mokrani and Madani showed that TPC yield in peaches decreased, when the temperature increased from 25 to 70 °C with values of 363 mg and 284 mg of GAE/100 g of dry weight, respectively [15].

Regarding the experimental ethanol concentrations, increased TPC content was observed with higher concentrations of ethanol, although extracting with 25% ethanol was not statistically different from extracting with 50% ethanol (Figure 1). In other words, up to an 82% increase of TPC was observed with 50% ethanol vs. aqueous extracts (no ethanol), at 60 °C and 2 h of extraction time. However, this trend was not as clear after 1 h of extraction at 40 and 50 °C, since 25% ethanol improved extraction of TPC, compared to those from 50% ethanol. In a similar way, this tendency was also observed at a temperature of 60 °C, with an extraction time of 3 h. Therefore, the use of ethanol in mixtures with water contributed towards a polar medium and improved the extraction of phenolic compounds.

The studies carried by Naczk and Shahidi [32], showed that the solubility of phenolic compounds depends on the polarity of solvent, as well as the formation of insoluble complexes, and the interaction with other components from foods. Yuan et al. used methanol, ethanol and acetone in concentrations of 20%, 50% and 80% for the extraction of TPC in the hazelnut shells [26]. The highest values of TPC were extracted with 50% of acetone. Socaci et al. showed that the most effective solvent for the extraction of TPC in beer by-products was acetone:water at 60% (*v*/*v*), with values of 114 mg GAE/100 g of dry weight [33]. Meneses et al. also obtained similar results in beer by-products, having value of 990 mg GAE/100 g of dry weight when 60% (*v*/*v*) acetone was used [34].

In another study, Mokrani and Madani evaluated the influence of the solvent type at different concentrations in the extraction of TPC from peach [15]. The optimal extraction was observed after using 60% acetone (*v*/*v*), with a value of 363 mg GAE/100 g of dry weight. This was as twice as high as the extraction made by water or with 60% ethanol (*v*/*v*), when it equaled to 182 mg and 178 mg GAE/100 g of dry weight, respectively. Tournour et al. reported the TPC from various grape marcs in a range of 6.93–13.17 mg GAE/100 g of dry residue, that were extracted with 80% aqueous ethanol (*v*/*v*) over 48 h [35]. Caldas et al., used ethanol in concentrations of 8%, 20%, 50%, 80%, and 92% for the extraction of TPC from grape skins [36]. Here, in the comparison to other concentrations, results showed a greater quantity of phenolic compounds for extracts with 50% ethanol (*v*/*v*).

The extraction time significantly influenced the amount of obtained TPC. The concentration of phenolic compounds increased with prolonged extraction, when using 25% ethanol (*v*/*v*) at T = 60 °C. For 1 h of extraction, the value was 7.22 mg GAE/100 g of dry matter, whereas for 2 h and 3 h they were 14.46 mg and 16.02 mg of GAE/100 g of dry matter, respectively. Studies conducted by Yuan et al., showed similar results after extracting TPC from hazelnut shells [26].

TPC obtained from tiger nut samples from Nigeria had values of 115.70 mM GAE/100 g of tiger nut in roasted tubers [37]. These values were higher, compared to those obtained in raw or dried nuts, and equaled to 80.70 mM GAE/100 g of chufa. Koubaa et al., used alternative methods for the extraction of TPC in tiger nuts’ oil [38]. The results obtained by these authors showed values of 4.53 mg and 6.21 mg GAE/100 g of oil for supercritical fluids (SC-CO_2_) extraction conducted at 20 and 30 MPa. Similar results were obtained after mechanical expression (ME), using similar treatment conditions, where obtained values were 4.71–5.29 mg GAE/100 g of oil. In this sense, Ezeh et al. also obtained values of 16.50 mg GAE/100 g of oil after applying SC-CO_2_ [39]. Mokrani and Madani, obtained the optimum TPC after 3 h extraction from the peach samples, and with a value of 363 mg GAE/100 g of dry weight. However, the use of longer times did not improve the extraction of phenolic compounds, indicating that too long of an extraction time can increase phenolic oxidation [15].

### 3.2. Total Flavonoids

**Figure 2:**
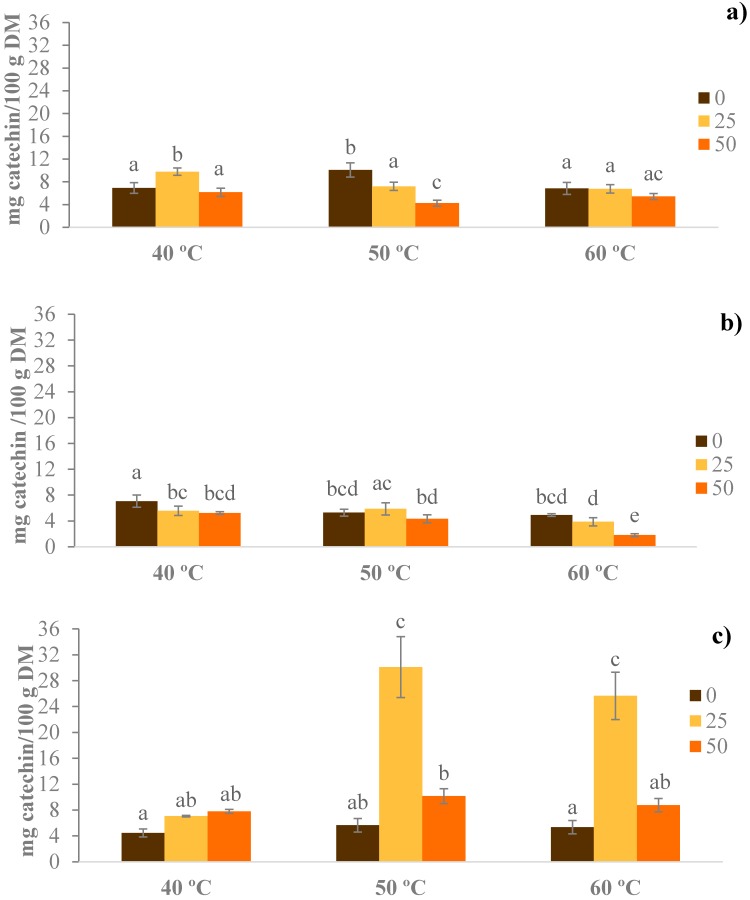
Total flavonoid content, after extraction with different hydroethanolic mixtures (0, 25 and 50% ethanol:water, *v*/*v*), temperature (40, 50 and 60 °C) and extraction time: (**a**) 1 h; (**b**) 2 h; and (**c**) 3 h. Different letters show statistically significant differences (*p* < 0.05) for temperatures and concentrations of ethanol used.

The content of TF in the extracts was between 1.80 and 30.09 mg CE/100 g of dry matter, depending of the used temperature, time, and concentration of ethanol. The optimal value of extraction was observed at 50 °C, with 25% of ethanol, and 3 h of extraction time. Both, the time and percentage of ethanol had a significant effect on the TF content, while temperature showed no influence (Figure 2). In general, higher values were with 25% of ethanol, compared to the mixtures without ethanol and with 50% ethanol. These results can be explained by different polarities of the solvent, since the mixtures of ethanol:water, have a greater polarity and therefore greater efficiency in the extraction of these components [31].

The researchers evaluated the effect of the solvent in determination of TF in beer by-products [33]. The highest values of TF was obtained after using a mixture of ethanol:water (80:20, *v*/*v*) (≈461 mg equivalents of quercetin (QE)/100 g of dry weight), compared to the aqueous extraction (2 mg QE/100 g of dry weight) under similar extraction conditions. Moreover, using ethanol in the range of those used in this study (40–60% and 60:40, *v*/*v*, ethanol:water), the recovery yield was ≈50 and 130 times higher than the aqueous extraction, respectively. Another study, showed that 60% ethanol (*v*/*v*) was the most efficient (57 mg QE/100 g of dry weight) for TF extraction from peach samples, obtaining values ≈3.3-fold higher, as compared to the aqueous extraction (17 mg QE/100 g of dry weight) [15]. Similar results for TF were obtained in beer by-products, with a value ≈50 mg QE/100 g of dry weight after using a mixture of ethanol:water (60:40, *v*/*v*) (≈50 mg QE/100 g of dry weight), obtaining ≈3-fold higher values, compared to the aqueous extraction under similar extraction conditions [33].

Regarding the influence of temperature on TF, a greater content of TF at 50 vs. 60 °C was observed, reaching an increase of up to 17% during 3 h of extraction (Figure 2). However, these results may be caused by higher temperatures that reduced the stability of these compounds. The studies on peach samples showed an increased yield of TF with the temperature increase from 25 to 60 °C, and values of 38 to 81 mg QE/100 g of dry weight [15]. However, temperatures above 60 °C reduced the value of TF in the samples.

In general, there is no clear trend in the results regarding the time for the extraction of TF. However, the extraction time of 3 h (25% ethanol (*v*/*v*); T = 50 °C) had the most TF, with values of 30.09 mg CE/100 g of dry matter, as compared to 7.20 and 5.87 mg CE/100 g of dry matter, after 1 and 2 h of extraction, respectively.

### 3.3. Total Antioxidant Capacity (TEAC)

Analysis of variance showed that temperature, ethanol percentage and time had a statistically significant effect (*p* < 0.05) on the total antioxidant capacity in the extracts.

**Figure 3:**
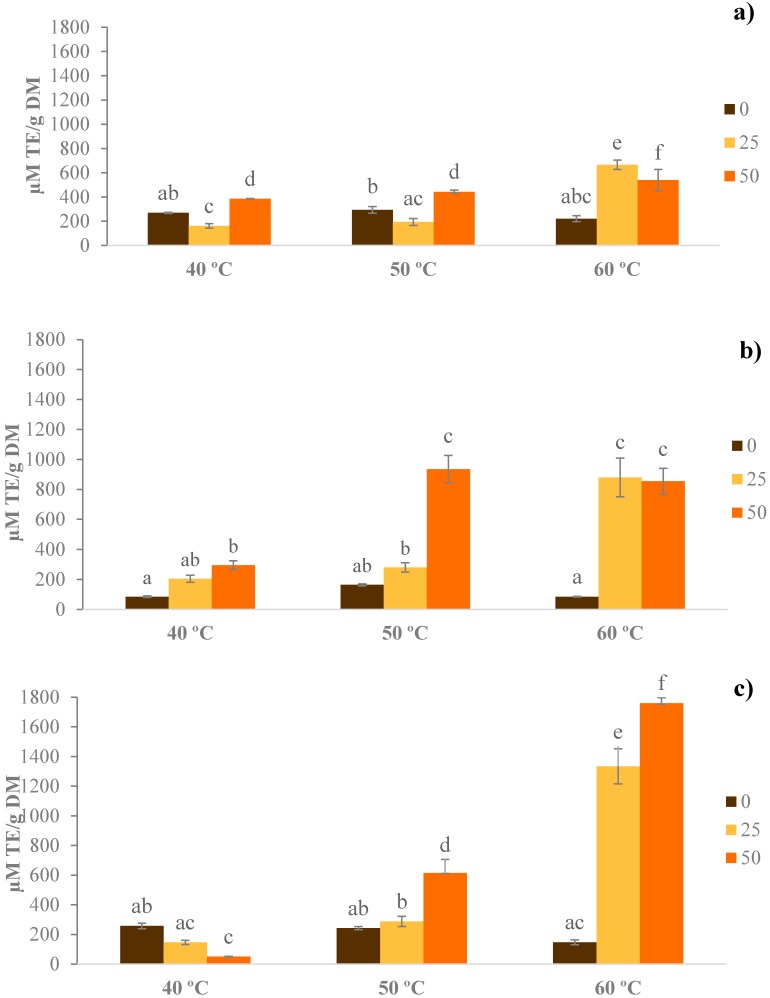
Total antioxidant capacity µM Trolox equivalents (TE)/g of dry matter (DM), after extraction with different hydroethanolic mixtures (0, 25 and 50% (*v*/*v*) ethanol:water,), temperature (40, 50 and 60 °C) and extraction time: (**a**) 1 h; (**b**) 2 h; and (**c**) 3 h. Different letters show statistically significant differences (*p* < 0.05) for temperatures and concentrations of ethanol used.

As seen in Figure 3, generally, the antioxidant capacity showed a tendency to increase with higher concentrations of ethanol. This may be due to the fact that the use of ethanol in mixtures with water created a more polar medium, thus, favoring the extraction of these compounds [34].

The highest values of TEAC (1759.81 µM Trolox/g of dry matter) were observed with the highest ethanol concentration (50%, *v*/*v*), longest extraction time (3 h), and temperature T = 60 °C.

The application of high temperature can favor the extraction of antioxidant compounds and increase the solubility.

### 3.4. Relationship between Total Antioxidant Capacity, Total Phenols and Total Flavonoids

To identify whether there was any correlation between total antioxidant capacities, total phenols and total flavonoids, a Pearson’s correlation analysis was performed (Table 1).

As it can be seen in Table 1, a strong positive correlation was observed between TEAC and TPC (*r* = 0.712, *p* < 0.05). Medium positive pairwise correlations were observed for TPC and the TF (*r* = 0.314, *p* < 0.05). However, the correlations between TF and TEAC were not significant (*r* = 0.194, *p* > 0.05). Similar results were obtained in peach extracts, where positive correlations between total phenolic compounds and antioxidant capacity (*r* = 0.76, *p* < 0.001) were reported. On the other hand, the correlation between TF and antioxidant capacity was negative (*r* = −0.82) [15].

The study by Socaci et al. showed a positive correlation between the DPPH antioxidant activity and the content of polyphenols/flavonoids in beer by-products [33]. Here, coefficients were much stronger and equaled to 0.908 for flavonoids, and 0.980 for phenolic compounds. Tournour et al. obtained similar results, with a positive correlation between phenolic compounds and DPPH method (*r* = 0.944) in wine by-products [35].

## 4. Conclusions

From the results of the present study, it can be concluded that by-products from the production of “horchata de chufa” are an important source of bioactive antioxidants. Moreover, temperature, time, and ethanol concentration presented a statistically significant influence on the recovery of total BACs. Higher temperatures increased the extraction efficiency of TPC, while in the same way, ethanol concentrations and prolonged extraction times improved TPC yields. Maximum extraction values of 16.02 mg GAE/100 g of dry matter were observed after using 25% ethanol (*v*/*v*), at 60 °C with an extraction duration of 3 h.

The concentration of ethanol and time showed a significant influence on the recovery of total flavonoids (*p* < 0.05). Generally, TF values were higher after using 25% ethanol (*v*/*v*), as compared to 50% of ethanol (*v*/*v*) and its complete absence. The TF values increased with temperature and reached the optimum value of 30.09 mg CE/100 g of dry matter at 50 °C, extracted with 25% ethanol (*v*/*v*) after 3 h of extraction.

A statistically significant influence of temperature and ethanol concentration was found for the recovery of antioxidant compounds (*p* < 0.05). In general, the antioxidant capacity will increase with higher concentrations of ethanol. This same trend was observed with respect to the time and temperature. The maximum yield of 1759.81 μM Trolox/g of dry matter was found with 50% ethanol (*v*/*v*), 60 °C and an extraction time of 3 h. TAEC and TPC had positive correlation similar to TPC and the TF. Further studies of by-products from the “horchata” should focus on the use of alternative methods for the extraction of antioxidant compounds aiming to reduce the time, usage of minimally toxic (in)organic solvents, and lower the processing temperatures.

## Figures and Tables

**Table 1:** Pearson’s correlation coefficients between antioxidant capacity (TEAC), phenolic compounds (TPC) and total flavonoids (TF).

	TPC	TF
**TEAC**	0.712 *	0.194
**TPC**	-	0.314 *

* Statistically significant correlations.
